# Discriminating between JCPyV and BKPyV in Urinary Virome Data Sets

**DOI:** 10.3390/v13061041

**Published:** 2021-05-31

**Authors:** Rita Mormando, Alan J. Wolfe, Catherine Putonti

**Affiliations:** 1 Bioinformatics Program, Loyola University Chicago, Chicago, IL 60660, USA; rmormando@luc.edu; 2 Department of Microbiology and Immunology, Stitch School of Medicine, Loyola University Chicago, Maywood, IL 60153, USA; awolfe@luc.edu; 3 Department of Biology, Loyola University Chicago, Chicago, IL 60660, USA

**Keywords:** JCPyV, BKPyV, polyomaviruses, urinary virome, urinary microbiome

## Abstract

Polyomaviruses are abundant in the human body. The polyomaviruses JC virus (JCPyV) and BK virus (BKPyV) are common viruses in the human urinary tract. Prior studies have estimated that JCPyV infects between 20 and 80% of adults and that BKPyV infects between 65 and 90% of individuals by age 10. However, these two viruses encode for the same six genes and share 75% nucleotide sequence identity across their genomes. While prior urinary virome studies have repeatedly reported the presence of JCPyV, we were interested in seeing how JCPyV prevalence compares to BKPyV. We retrieved all publicly available shotgun metagenomic sequencing reads from urinary microbiome and virome studies (*n* = 165). While one third of the data sets produced hits to JCPyV, upon further investigation were we able to determine that the majority of these were in fact BKPyV. This distinction was made by specifically mining for JCPyV and BKPyV and considering uniform coverage across the genome. This approach provides confidence in taxon calls, even between closely related viruses with significant sequence similarity.

## 1. Introduction

High-throughput sequencing technologies have allowed us to discover the complex communities of bacteria and viruses that inhabit the human body: the human microbiota. In contrast to many organ systems, the urinary tract of asymptomatic individuals was once thought to be “sterile.” This belief was primarily based on the observed absence of bacterial species and was recently debunked using high-throughput sequencing (see review [1]). In the past, the urinary tract was referred to as sterile despite the well-accepted knowledge that viruses are shed into urine. It has been 50 years since polyomaviruses were reported within the urinary tract [2].

Polyomaviruses are the smallest known double-stranded DNA viruses and are abundant in the human microbiota [3]. Within the urinary tract, two polyomaviruses have been identified: JC virus (JCPyV) and BK virus (BKPyV). The genomes of these two viruses are 75% similar, encoding for the same six proteins. In most cases, JCPyV and BKPyV are considered benign members of the urinary microbiota, producing persistent, asymptomatic infections of the kidneys [4].

Estimates of JCPyV prevalence within the population range from 20 to 80% [5,6,7]. Studies have found that the incidence of JCPyV is low in younger populations and high in the elderly [5,8]. The estimates of prevalence are based upon amplification-based surveys, and numerous assays/protocols have been designed to detect JCPyV and BKPyV, including distinguishing between the two [9,10,11,12,13,14,15,16,17,18,19,20,21,22,23,24,25,26,27,28,29,30,31,32]. Although currently there are just a handful of metagenomic and viromic studies of the urinary microbiome, JCPyV and/or BKPyV have frequently been reported [33,34,35,36,37]. Previously, we were able to assemble the complete JCPyV genome from the urinary microbiomes of five different women [35]. This prompted our investigation of JCPyV in the urinary microbiome data sets.

## 2. Materials and Methods

Publicly available raw reads from metagenomic sequencing of the urinary microbiome were identified from the literature [33,36] and retrieved from NCBI’s Short Read Archive (SRA) and iMicrobe (summarized in Table 1, full details in Table S1). We also included 4 samples sequenced from portable urinals. These publicly available data sets, as well as the urinary microbiome generated as part of this study, were processed as follows: each data set was mapped to a reference genome using Bowtie2 [38] through either Geneious Prime (v2.3.2; Biomatters, Ltd., Auckland, NZ) or from the stand-alone application (v.2.3.5). Reference genomes included the JCPyV RefSeq (accession no. NC_001699.1) and the BKPyV RefSeq (accession no. NC_001538.1).

Additionally, we sequenced the urinary microbiome of one urine sample in our collection, which was confirmed to be PCR-positive for JCPyV. This urine was collected via transurethral catheterization from a woman with overactive bladder as part of a previous IRB-approved study [39]. DNA was extracted using a phenol–chloroform protocol with a starting volume of 500 uL urine. The extracted DNA was sent to the Microbial Genome Sequencing Center (Pittsburgh, PA, USA). Libraries were prepared using a method based upon the Illumina Nextera kit (Illumina, Inc., San Diego, CA, USA) and sequenced on the Illumina NextSeq 550 platform. Sequencing produced 93.5 M reads of read length 150 nucleotides. Raw reads have been deposited in SRA; accession number SRR13199001.

## 3. Results

**Figure 1:**
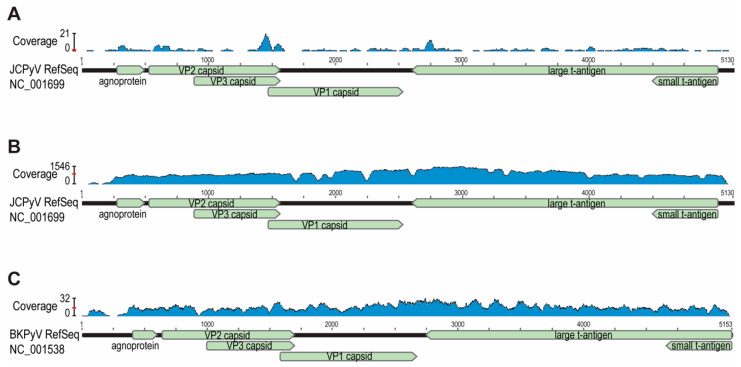
Raw read coverage of JCPyV and BKPyV reference genomes. (**A**) Coverage of the JCPyV genome for sample ERR2798128 reads; average coverage (indicated by red bar) was ~1 reads/nucleotide and was concentrated on sequences conserved between JCPyV and BKPyV. (**B**) Coverage of the JCPyV genome for sample ERR2798125 reads; average coverage (indicated by red bar) ~900 reads/nucleotide. (**C**) Coverage of the BKPyV genome for sample ERR2798128 reads; average coverage (indicated by red bar) was ~15 reads/nucleotide.

In an effort to ascertain the frequency at which JCPyV is detectable within the urinary microbiome, we retrieved all publicly available urinary metagenomic data sets (Table S1). Included in these data sets were samples in which the corresponding published paper indicated that JCPyV was present based upon microbiome sequencing data alone [33,36]. Of the 165 data sets processed, raw reads mapped to the JCPyV RefSeq sequence (5130 base pairs (bp)) for 59 of the samples (minimum number of reads mapped: 1; maximum number of reads mapped: 336,004; average number of reads mapped: 36,734). Further investigation of the mapped reads, however, revealed that the genome coverage of these reads was unequal; particular genes were mapped with significantly high coverage, whereas others had few or no reads mapped to them (Figure 1A). In stipulating that mapped reads must be uniformly distributed across the JCPyV genome (Figure 1B), we conclude that with high confidence JCPyV is present in only three samples: accession nos. ERR2798125, ERR2798126, and SRR6519218. The first two samples are from the Kidney Transplant Virome data set [34] and the third is from the Pulmonary Tuberculosis Urinary Microbiome data set. For the 56 data sets failing this uniformity test, we mapped the raw reads against the BKPyV RefSeq sequence (5153 bp), finding uniform coverage (Figure 1C). Thus, we can conclude that JCPyV is present in only 2% of the samples tested.

Next, we conducted shotgun sequencing for one PCR-positive urine sample to confirm that JCPyV could be detected in the urinary metagenome. One pair of reads (of the 46.7 million pairs) was mapped to the JCPyV RefSeq. These reads were then queried against the nr/nt database via blastn and had 100% query coverage (length 150 bp) and 100% sequence identity to JCPyV sequences. Both reads map to the large t-antigen coding region. These reads were also compared to BKPyV sequences (Human Polyomavirus 1, taxid: 1891762) via discontiguous blast. The two reads had 99% query coverage and 75% sequence identity and 60% query coverage and 80.22% sequence identity, respectively. This suggests that these reads are in fact representative of JCPyV and not BKPyV.

## 4. Discussion

This study provides insight into the challenges of definitively identifying JCPyV in urinary microbiomes/viromes amidst other polyomaviruses, namely BKPyV. Making this distinction is paramount if we are to accurately determine the prevalence of JCPyV in the population and begin to explore what role (if any) JCPyV plays in urinary health.

Metagenomic studies of urine samples have repeatedly suggested that JCPyV and BKPyV are both present [33,34,35,36,37]. However, our analysis of 165 publicly available urinary data sets suggests that BKPyV may be mistakenly reported as JCPyV. It is important to note that the data sets examined here (Table 1) were not explicitly looking for JCPyV. Similarly, in our own prior urinary microbiome study [35], we were not focused on identifying JCPyV. The current estimates of JCPyV vary widely, from 20 to 80% [5,6,7]. This estimate comes from PCR-based or serotype-based assays and the variation reported is largely due to differences in the populations sampled (e.g., age, sex). It is important to note that the metagenomic data sets examined here cannot provide any insight into JCPyV prevalence as they also differ in the populations sampled, as well as in methods of sample collection (e.g., catheterized urine, voided urine, pooled urine samples) and DNA extraction.

Bioinformatic tools designed for analysis of large high-throughput studies often lack the sensitivity required to differentiate between closely related species, a trade-off made in favor of speed. Tools designed to recognize viral sequences in metagenomic data sets often rely on the presence of hallmark genes or best BLAST hits to make taxonomic calls [40,41,42,43,44,45]. Given the high sequence homology between BKPyV and JCPyV, miscalls of the two polyomaviruses are not surprising. Here, we have shown that these distinctions can only be reliably made by considering each species independently, examining the evenness of read coverage as well as coverage of and homology to unique regions. For rare members of the virome, analysis at the read level may even be necessary. For instance, the urinary metagenome produced as part of this study, which was PCR positive for JCpyV, included two reads to JCPyV. These reads exhibit greater homology to JCPyV than to BKPyV sequences.

Thus, while metagenomic sequencing has significant potential for virus detection, rigorous bioinformatic interrogation is necessary to correctly identify closely related species. Similar to our approach, others have specifically mined metagenomes/viromes in an effort to distinguish between closely related species of interest, e.g., HPV [46,47]. Based upon our study here, we advocate that evenness of read coverage should be considered as a finishing step in any virome analysis, as it provides confidence in taxon calls.

## Figures and Tables

**Table 1:** Summary of raw read data sets evaluated in this study.

Study	Accession No.	# Samples	# Reads Total
Kidney Transplant Virome [34]	PRJEB28510 ^1^	27	157.5 M ^3^
Pulmonary Tuberculosis Urinary Microbiome (unpublished)	PRJNA431965 ^1^	3	9.5 M ^4^
Microbial Metagenome of UTI [36]	PRJNA385350 ^1^	49	532.0 M ^4^
Virome in Healthy and BK Disease of Kidney Transplant (unpublished)	PRJNA587166 ^1^	62	70.0 M ^3^
Virome in Association with UTI [33]	cobian9680 ^2^	20	14.5 M ^3^
Portable Urinal Microbiome (unpublished)	PRJNA399057 ^1^	4	228.3 M ^4^

^1^ Accession number for NCBI BioProject (https://www.ncbi.nlm.nih.gov/bioproject); ^2^ Accession number for iMicrobe (https://www.imicrobe.us); ^3^ Sequencing performed for viral community only; ^4^ Sequencing performed for bacterial and viral community.

## Data Availability

Raw reads for the urinary metagenome produced in this study have been deposited in SRA; accession number SRR13199001.

## Supplementary Materials

The following are available online at https://www.mdpi.com/article/10.3390/v13061041/s1, Table S1: Publicly available metagenomes mined for JCPyV.
